# Amidated Pluronic Decorated Muco-Penetrating Self-Nano Emulsifying Drug Delivery System (SNEDDS) for Improved Anti-*Salmonella typhi* Potential

**DOI:** 10.3390/pharmaceutics14112433

**Published:** 2022-11-10

**Authors:** Rabia Arshad, Muhammad Salman Arshad, Tanveer A. Tabish, Syed Nisar Hussain Shah, Saira Afzal, Gul Shahnaz

**Affiliations:** 1 Faculty of Pharmacy, The University of Lahore, Lahore 54000, Pakistan; 2 Hamdard College of Medicine and Dentistry (HCMD), Karachi 74600, Pakistan; 3 Radcliffe Department of Medicine, University of Oxford, Oxford OX3 7BN, UK; 4 Department of Pharmacy, Quad-i-Azam University, Islamabad 45320, Pakistan

**Keywords:** amidated pluronic, mucopenetration, biofilm dispersion, anti-salmonella potential

## Abstract

The enteric system residing notorious *Salmonella typhimurium* (*S. typhi*) is an intracellular, food-borne, and zoonotic pathogen causing typhoid fever. Typhoid fever is one of the leading causes of mortality and morbidity in developing and underdeveloped countries. It also increased the prevalence of multidrug resistance globally. Currently, available anti-bacterial modalities are unable to penetrate into the intracellular compartments effectively for eradicating *S. typhi* infection. Therefore, in this study, we developed nanostructured lipid-based carriers in the form of a self-nanoemulsifying drug delivery system (SNEDDS) for targeted delivery of ciprofloxacin (CIP) into the *S. typhi* intracellular reservoirs. Capryol 90, Tween 80, and Span 20 were finalized as suitable oil, surfactant, and co-surfactant, respectively, according to the pseudoternary phase diagram emulsifying region. Targeting capability and mucopenetration of the SNEDDS was attributed to the inclusion of amidated pluronic (NH_2_-F_127_). Developed NH_2_-F_127_ SNEDDS were characterized via physicochemical, in vitro, ex vivo, and in vivo evaluation parameters. The size of the SNEDDS was found to be 250 nm, having positively charged zeta potential. In vitro dissolution of SNEDDS showed 80% sustained release of CIP in 72 h with maximum entrapment efficiency up to 90% as well as good hemocompatibility by showing less than 0.2% hemolysis and 90% biocompatibility. The survival rate of *S. typhi* in macrophages (RAW 264.7) was minimal, i.e., only 2% in the case of NH_2_-F_127_ SNEDDS. Macrophage uptake assay via nanostructures confirmed the maximum cellular uptake as evidenced by the highest fluorescence. Biofilm dispersion assay showed rapid eradication of developed resistant biofilms on the gall bladder. In vivo pharmacokinetics showed improved bioavailability by showing an increased area under the curve (AUC) value. Taken together, NH_2_-F_127_-SNEDDS can be utilized as an alternative and efficient delivery system for the sustained release of therapeutic amounts of CIP for the treatment of *S. typhi.*

## 1. Introduction

*Salmonella typhi* (*S. typhi*) is considered a major zoonotic pathogen worldwide, causing enormous loss of livestock as well as public health. *S. typhi* is the etiological reason behind gastroenteritis and life-threatening typhoid fever, thereby imposing a major socioeconomic burden globally [1]. *S. typhi* invades intestinal epithelial M-cells via its locomotive pili and resides in the intestinal issues, followed by the dissemination into the systemic circulation upon host inflammatory response towards the pathogen. *S. typhi* survives under harsh conditions owing to its penetration ability and widespread replication into the reticuloendothelial system (RES), presenting symptoms such as hyperthermia, headache, body aches, and severe diarrheal condition [2]. Antimicrobial agents have been found to markedly reduce the mortality rate, but some resistant pathogenic strains are causative agents for multi-drug resistance by blocking or modifying targeted sites of action. Although numerous antimicrobial agents present effective treatment against infectious diseases, most of them lack potency against multi-drug resistant pathogens. Moreover, in the case of *S. typhi* treatment, fluoroquinolones (ciprofloxacin) are the most commonly used and preferable class of medications. However, existing antimicrobials such as CIP belong to the biopharmaceutical classification system (BCS) IV, having low solubility and permeability. CIP showed low bioavailability, having a half-life of 3–4 h. Hence, owing to these physicochemical and pharmacokinetic features, CIP is incapable to reach RES reservoirs, leading to a non-uniform and unspecified drug delivery [3]. Targeted delivery to reach intestinal reservoirs for the eradication of *S. typhi* highly demands an oral formulation having the ability to overcome mucosal barriers in response to maximum penetration and uniform distribution [4]. Pathogenic diseases like *S. typhi* treatment have been modernized by the embarking of nanotechnology in drug delivery. Nanoparticles also have the capability to combat bacterial resistance via novel techniques such as magnetism by forming electrostatic attraction between negatively charged bacterial cells and positively charged magnetic nanoparticles (NP+), for capturing bacteria.

Polymeric, inorganic, metallic, and lipid nanoparticles are most important in this regard. Lipid nanoparticles are exceedingly adept in averting the solubilization and absorption issues of anti-bacterial drugs used in *S. typhi* treatment in terms of controlling drug release. Moreover, CIP and other anti-microbial drugs can be proficiently reached by the targeted site when conjugated with lipid-based nanoparticles such as liposomes, niosomes, solid lipid nanoparticles, core–shell nanoparticles, and SNEDDS. Various nanoformulations can be developed by entrapping the CIP to induce proficient antibiotic delivery. In this case, fucoidan-coated and CIP-loaded chitosan nanoparticles were developed by S. Elbi and co-workers to determine the anti-salmonella potential [5]. Similarly, in another research, Rajeev J. Mudakavi and co-workers synthesized arginine-coated nanocarriers for CIP delivery in the macrophages for targeted action [6]. However, SNEDDS are preferential as compared to the previously synthesized nanocarriers in terms of improved bioavailability and reduced dose frequency.

SNEDDS are isotropic mixtures of drugs, and suitable excipients (oil, surfactant, and co-surfactant) have the capability to generate ultra-fine nanoemulsion when coming in contact with gastrointestinal fluid contents, thus leading to maximum absorption. Moreover, they are cost-effective, easy to manufacture, and possess the capability to deal with both hydrophilic and hydrophobic drugs [7].

However, functionalization of the SNEDDS with suitable polymer or ligand is important in achieving desirable outcomes.

Here in this study, we developed nanostructured lipid-based carriers in the form of a self-nanoemulsifying drug delivery system (SNEDDS) for targeted delivery of CIP into the *S. typhi* intracellular reservoirs [8]. Antibacterial activity and muco-penetration of the SNEDDS were endorsed via the inclusion of amidated pluronic (NH_2_-F_127_) [9,10]. NH_2_-F_127_ was synthesized in two steps involving initial conversion into an aldehyde group and then into cationic amines to ensure maximum thermodynamic stability [11]. NH_2_-F_127_ functionalization leads to more advantages as compared to the already synthesized nanoemulsion in terms of improved mucoadhesion, mucopenetration, safety profile, and thermodynamic stability. The antibacterial mechanism of pluronic-amines is a remarkable paradox as the introduction of the cationic NH group leads to increased intracellular penetration via improved junction and targeted release in the *S. typhi* to diminish the bacterium.

## 2. Materials and Methods

### 2.1. Materials

CIP, potassium hydroxide (KOH), acetic anhydride, DMSO, DCM, sodium cyanoborohydride (NaBH_4_), oils, surfactants, co-surfactants, pluronic 127, culture media, fluorescent dyes, and Schiff reagent were obtained from Sigma-Aldrich, Germany. All solvents used were of analytical grade.

### 2.2. Methods

#### 2.2.1. Synthesis of Amidated Pluronic (NH2-F127)

Polyethylene oxide modifications with different functional groups have various biomedical applications. Similarly, pluronic is the repetition of polyethylene oxides and the conversion of the pluronic into pluronic-amines. In step 1, 0.5 mmol of pluronic was dissolved in the DMSO solution, followed by the addition of acetic anhydride in the reaction mixture with stirring at room temperature for 30 h to develop pluronic aldehyde. Afterward, the reaction was stopped by the addition of cold di ethyl ether. Schiff reagent test confirmed the formation of aldehyde groups by indicating rose color. After the confirmation of aldehydic group in poloxamer, the reaction mixture was switched toward the second step. An amount of 0.20 mmol pre-aldehyde mixture was added with 4 mmol NH_4_Cl and 0.02 g KOH in 5 mL methanol. Furthermore, 4 mmol sodium cyano borohydride (NaBH_4_) in 5 mL methanol was added to the preformed solution gradually. Moreover, the reaction mixture was mixed with 0.1 g KOH and stirred for 24 h at room temperature and then precipitated with cold ether and filtered [12]. However, the final filtered polymer was then dialyzed using double distilled water followed by lyophilization.

#### 2.2.2. Quantification of Primary Amines

Calorimetric 2,4,6-tri nitro benzene sulfonic acid (TNBS) assay was performed to determine the primary amine functional groups in the amidated pluronic at each level of modification. Therefore, 0.5 mg of the polymer was dissolved in 500 μL NaCl (0.5%, *w*/*v*) solution followed by incubation at 25 °C for 30 min. Furthermore, 500 μL of TNBS (0.1%, *w*/*v*) containing NaHCO3 (4%, *w*/*v*) was incorporated into each hydrated aliquot followed by further incubation for 3 h and centrifugation for 5 min at 4 °C. Moreover, absorbance was measured using a microtiter plate reader [13].

#### 2.2.3. Solubility Testing of Excipients

CIP solubilization was determined via shake flask method, in which a broad range of above-mentioned excipients was evaluated. In this study, excess of drug was dissolved in approximately 2 mL of each excipient through vortex mixing for 5 min. Afterward, the mixture was placed in shaking incubator with ambient heating for 24 h to develop optimized SNEDDS [14].

#### 2.2.4. Pseudoternary Phase Diagram

Pseudoternary phase experimental design was formulated in CHEMIX School software on the basis of differences in the ratios of oil, surfactant, and co-surfactant showing accumulative 100% excipients ratio [15,16].

#### 2.2.5. Formulation of the NH2-F127 Loaded Polymeric SNEDDS

Simple SNEDDS formulation can be formulated by adding 25 mL (CIP) drug, Capryol oil (45%), Tween 80 (30%), and Span 20 (25%). However, by mixing suitable quantities of NH2-F127 polymeric conjugate (5%) along with drug, oil, surfactant, and co-surfactant in sonicator for 10 min at 55 °C polymeric SNEDDS were developed. Ratio of the excipients was adjusted according to the prominent nanoemulsion region of pseudoternary phase diagram.

### 2.3. Size, Poly Dispersity Index (PDI), Zeta Potential, and SEM Analysis

SNEDDS formulation was diluted with distilled water, and vortex mixed for 1 min. Final formulation obtained after probe sonication was termed nanoemulsion and it was evaluated for determining size, zeta potential, and PDI through zeta sizer (ZS90, Malvern Instrument, London, UK). Moreover, scanning electron microscopy (SEM) analysis was performed for determining surface morphology [17].

### 2.4. Physicochemical Characterization Tests

#### 2.4.1. Transmittance, Dispersibility, and Thermodynamic Stability Testing

Double beam spectrophotometer (Halo DB-20, Dynamica, London, UK) was used to determine the percentage transmittance (%) of SNEDDS dilutions at 276 nm [17]. To evaluate dispersibility of the SNEDDS, 1 mL of the SNEDDS was diluted in 100 mL of deionized water and mixed thoroughly at ambient temperature using magnetic stirrer at 50 rpm. Dispersibility test is based on the duration required to convert milky emulsion into clear one [18]. Thermodynamic stability evaluation of the SNEDDS was completed in two phases, i.e., centrifugation and temperature stress. Initially, SNEDDS were tested by centrifugation for 45 min at 13,500 rpm, using a centrifuge machine. Afterward, the SNEDDS were exposed to harsh temperature conditions, i.e., freezing (−20 °C) and heating (50 °C) through incubation at 4 °C, followed by storage at 25 °C for observation of any visible signs of instability [19].

#### 2.4.2. Cloud Point Temperature Measurement

The temperature needed to convert the clear nanoemulsion into cloudy one is known as cloud point temperature measurement. For this purpose, SNEDDS (1 mL) after 100 times dilution in the deionized water were stirred on hot plate magnetic stirrer with regular temperature augmentation, till the conversion into cloudy emulsion [20].

#### 2.4.3. Robustness of Dilution

Robustness to dilution is basically completed to determine the capacity of the SNEDDS to endure dilution stress. For this purpose, dilution of the SNEDDS in the multiples of 10 (1:10, 1:100, 1:1000) in deionized water, phosphate buffer saline (PBS), and simulated intestinal fluid (SIF) was completed, followed by storage for 24 h to determine any stability changes [21].

#### 2.4.4. Fourier Transformed Infrared Spectroscopy (FTIR) and Differential Scanning Calorimetry (DSC)

The electrostatic interaction among CIP and polymers in the SNEDDS was determined via FTIR analysis (Bruker α, Billerica, MA, USA). The KBr disk method was utilized to compress the powdered forms of CIP and polymers, followed by scanning of spectra in the range of 500–4500 cm^−1^ wavenumber. NMR can also be helpful in the determination of functional groups. The ^1^H NMR spectra of pluronic-amines have already been reported by Gyulai et al. (2016). The authors observed a strong singlet at 3.65 ppm that was attributed to CH_2_ groups of PEO blocks. Similarly, presence of CH-CH_2_- protons was indicated by two multiplets within the range of 3.3–3.6 ppm whereas, a peak observed at 1.14 ppm was related to the presence of PPO methyl group. Moreover, a broad signal appearing at 2.434 confirmed the presence of terminal amino group.

DSC was determined to probe the difference in the heat flow after modifying the backbone of polymer. For this purpose, samples were analyzed through differential scanning at a 50–300 °C temperature range at a specified rate under 50 KPa atmospheric nitrogen pressure.

### 2.5. In Vitro Drug Release Study

A simple diffusion method was followed to determine the drug release pattern of CIP from SNEDDS. For this purpose, NH2-F127 loaded SNEDDS of CIP dissolution were evaluated for 72 h at pH 5.0 for mimicking the endosomal environment. For this purpose, SNEDDS with 25 mg of CIP concentration were mixed with 100 mL PBS (pH 5.0) and placed in shaking water bath with 150 rpm at 37 °C. The pH 5.0 opts as it is the phagosomal pH and the purpose of this study is to deliver drug directly into the phagosomes. Moreover, due to hydrophobicity of CIP, it was sparingly soluble in PBS. To overcome the issue of CIP solubility and to enhance the redox-triggered release in the phagosomes, approximately 2 drops of 15 mM glutathione were added. Afterward, 2 mL of formulation was extracted at specified time intervals The extracted SNEDDS formulations were centrifuged at 13,500 rpm for 45 min at specific time points. The supernatant obtained after centrifugation was quantified for CIP release and pellet was redistributed with the same amount of fresh media, followed by the replenishment of same dissolution media. Furthermore, obtained CIP was quantified at 276 nm with UV visible spectrophotometer [22].

### 2.6. Drug Entrapment Efficiency

Initially, well-dispersed nanoemulsion was formed by dispersing small quantity of SNEDDS in the 5 mL de-ionized water with the help of 1 min probe sonication. CIP was separated from the nanoemulsion through ultracentrifugation at 13,500 for 30 min. However, the content of the drug was evaluated at 276 nm through a UV–Visible double beam spectrophotometer [4].

The following equation can be used to ascertain EE%:

EE (%) = 𝑊𝑡/𝑊𝑥 × 100(1)

where *Wt* = total amount of CIP within the prepared SNEDDS; *Wx* = total amount of CIP added for the SEDDS formulation.

### 2.7. Mucopenetration Study

Intestinal muco-penetration of the goat intestinal mucus was evaluated through silicone tube. Initially, goat mucus was separated and purified. Afterward, standard curves for the fluorescein di acetate were developed, followed by labeling of SNEDDS. Silicon tube one end was filled with the clean mucus and sealed, and the other open end was filled with the FDA-labeled SNEDDS. All formulations incorporated silicon tubes were incubated at room temperature with continuous stirring and freeze at −25 °C for 12 h. After freezing, numerous slices with 2 mm range were prepared. Hydrolysis of the FDA to sodium fluorescein was completed by adding of NaOH (500 µL) in each frozen slice along with incubation and ultra-sonication. Finally, reading was noted at 490 nm using plate reader for detecting the amount of solubilized fluorescein [22].

### 2.8. Mucoadhesion Study

The mucoadhesion of the SNEDDS was estimated through rheological synergism using cone plate viscometer. For this purpose, 5 mL artificial mucin solution was prepared and then 1 mL of nanoemulsion was immersed in the 5 mL artificial mucin solution and incubated at ambient temperature [23]. Rheology can be evaluated at the rate of 60 s by using the formula given below in Equation (2).

Δη = ηmix − ηmuc(2)

ηmix is rheology of mucin+ nanoemulsion and ηmuc is the rheology of mucin

### 2.9. Hemocompatibility Study

A hemocompatibility study was executed by taking 10 mL of human blood samples from healthy volunteers. After collection, the blood was mixed with normal saline and diluted with PBS in the fixed ratio of 1:9. SNEDDS formulations with varying concentrations in the range of (1–4 mg/mL) were added into the diluted blood and PBS mixture. PBS and blood mixture were considered as negative control. Blood and Triton X-100 mixture was considered as positive control. All these samples were incubated for 24 h, followed by centrifugation at 3000 rpm for 5 min at 4 °C to collect supernatant [24]. % Hemolysis can be calculated by following Equation (3):

(3) $$\%\;\mathrm{hemolysis}=\frac{\mathrm{Sample}\;\mathrm{absorbance}\;-\;\mathrm{negative}\;\mathrm{control}\;\mathrm{absorbance}}{\mathrm{Positive}\;\mathrm{control}\;\mathrm{absorbance}-\;\mathrm{negative}\;\mathrm{control}\;\mathrm{absorbance}}\;\times 100$$

### 2.10. In Vitro Lipolysis Studies

In vitro lipolysis studies were performed using a thermostat water bath containing lipolysis buffer. Afterward, SNEDDS were immersed in varying concentrations from 0.25–2 g in the above-mentioned lipolysis buffer. Equilibrium of the thermostat was maintained at 37 °C for 10 min. once equilibrium was maintained, process of digestion was initiated by adding lipase enzyme (4 mL) in the medium. Titration of the medium with NaOH was performed for maintaining the 7.4 pH by a pH stat. After digestion, process of ultracentrifugation started at 40,000 rpm for 2 h at ambient temperature, followed by pipetting of undigested lipids. However, the aqueous phase was separated and dissolved in DMSO. Furthermore, CIP quantification was completed by means of HPLC [23].

### 2.11. S. typhi Cytotoxicity in Macrophage RAW 264.7 Cells

*S. typhi* strains were cultured in the LB broth and macrophages were inoculated and incubated for 4 h at 37° C. RAW 264.7 cells were further nourished through sterilized DMEM media in combination with 10% (*v*/*v*) FBS and 1% antibiotics and placed in CO_2_ incubator at ambient temperature. Diluted SNEDDS formulations in the concentration ranges of 3.125–100 µg/mL were immersed into the DMEM media and placed in CO_2_ incubator at ambient temperature for 24 h. Culture media was further replaced and replenished with the fresh media containing 500 µg/mL MTT/PBS solution and incubated for 4 h. As a result, violet-colored formazan crystals were developed. For dissolving of the formazan crystals, 100 µL DMSO was added to each well, and absorbance was evaluated by multiplate reader at 540 nm. Graph pad Prism 8.02 software was used to compute IC_50_ values for each formulation [5].

### 2.12. Anti-Bacterial Activity

The antibacterial activity of SNEDDS formulations was examined by means of incubation with bacterial cell suspensions in PBS buffer (10 mM, pH 7.4). Afterward, 3 × 104 cells mL^−1^ concentration of *S. typhi* strains was mixed with polymeric SNEDDS. *S. typhi* was cultured in LB broth aerobically at 37 °C for 24 h with 120 rpm shaking. The minimum bactericidal concentrations (MBC) of F127 and NH2-F127 were determined using previously developed methods. The sterilization rate was also determined by the following equation:

Sterilization rate % = (C_0_ − C)/C_0_ × 100 (4)

where C is the CFU of the experimental group treated with CIP and polymeric SEDDS and C_0_ is the CFU of the control group receiving no treatment.

### 2.13. SNEDDS Uptake Studies

A fluorescent microscope (Evos^®^ FL Cell Imaging system, Thermo Fisher Scientific, Waltham, MA, USA) was used to evaluate the uptake of SNEDDS through macrophages RAW 264.7. Therefore, macrophages were planted onto the well plates maintaining 5000 cells/well density in provision with DMEM media in combination with 10% (*v*/*v*) FBS and 1% antibiotics. The seeded and media-nourished cells were then placed in CO_2_ incubator at 37 °C for achieving 50–60% confluency. Rhodamine-labeled SNEDDS were added to the wells for 4 h to achieve maximum internalization. Afterward, cells were rinsed with PBS for eradicating un-internalized SNEDDS. At the end, fluorescent microscopy was performed [24].

### 2.14. Biocompatibility Studies

Biocompatibility of SNEDDS formulations was determined by MTT assay. For MTT assay, macrophages were harvested in the well plates to achieve the density of 5000 wells. RAW 264.7 cells were further nourished through sterilized DMEM media in combination with 10% (*v*/*v*) FBS and 1% antibiotics and incubated in CO_2_ incubator at ambient temperature. Diluted SNEDDS formulations in the concentration ranges of 3.125–100 µg/mL were immersed into the DMEM media and placed in CO_2_ incubator at ambient temperature for 24 h. DMEM media was further changed and replenished with the fresh media containing 500 µg/mL MTT/PBS solution and incubated for 4 h. As a result, violet-colored formazan crystals were developed and finally dissolved by 100 μL DMSO and absorbance was noted at 540 nm.

% cell viability was calculated using the following Equation (5):

(5) $$\%\;\mathrm{cell}\;\mathrm{viability}=\frac{\mathrm{Absorbance}\;\mathrm{of}\;\mathrm{the}\;\mathrm{sample}-\mathrm{Absorbance}\;\mathrm{of}\;\mathrm{the}\;\mathrm{blank}}{\mathrm{Absorbance}\;\mathrm{of}\;\mathrm{the}\;\mathrm{control}-\mathrm{Absorbance}\;\mathrm{of}\;\mathrm{the}\;\mathrm{blank}}$$

### 2.15. Biofilm Elimination Assay

*S. typhi*-resistant strains were cultured in the nutrition media LB broth and placed in the polystyrene 12 well plates at ambient temperature for being incubated for two days. After the two days’ duration, SNEDDS were immersed into the culture well plates, to be incubated for further two days at ambient temperature. Afterward, the incubated plates were rinsed three times with PBS for removing unattached extracellular bacteria. Removal of extracellular bacteria was followed by the drying of plates via overnight inversion at ambient temperature. Drying was followed by staining of developed dried biofilms with 1% crystal violet, and staining was rinsed through distilled water. Seventy percent isopropyl alcohol was used for extraction of the crystal violet which remains bound to the biofilms, by measuring optical density at 595 nm. For the purpose of fluorescent imaging, biofilms were labeled with DAPI dye for observing the growth inhibition behavior of *S. typhi* biofilms through NH2-F127 [25].

### 2.16. Biofilms Dispersal Assay in Gall Stones

*S. typhi* has the capability to penetrate gall bladder and develop resistant biofilms on the gall stones. Therefore, gallstone infection impregnation is an important parameter to evaluate. Therefore, samples were collected with ethical permission from the administration of University of Lahore Teaching Hospital (UOLTH), Lahore, Pakistan. Gallstones were added to the LB broth cultured plates, followed by the *S. typhi* inoculation for two days to develop biofilms. Afterward, gallstones were rinsed with PBS and fixed with paraformaldehyde followed by drying and imaging via SEM [26].

### 2.17. In Vitro Survival Assay

In vitro survival rate of *S. typhi* was determined in the macrophages by culturing *S. typhi* strains in LB broth media, followed by the inoculation of DMEM media and 10% (*v*/*v*) FBS. Afterward, the macrophages were inoculated, and incubation was completed for 4 h at 37 °C, followed by the internalization of *S. typhi* into macrophages. Extracellular attached infected macrophages were treated with 1% antibiotics to eradicate further impurities. Washing with PBS was performed again to remove antibiotics.

### 2.18. In Vivo Pharmacokinetics

High-performance liquid chromatography (HPLC) method was used to detect the CIP levels after administration of SNEDDS in the healthy rabbits. HPLC analysis of the CIP at 278 nm was performed by using Shimadzu instrument of series 200, Japan, having column C18 and a length of 4.6 mm along with 150 mm width having 5 µm pore size. Two percent acetic acid and acetonitrile in the ratio of 84:16 *v*/*v* were used to develop mobile phase. Flow rate was set at 1.0 mL/min with injection volume of 20 µL. Animal study was approved by the Bioethical Committee of Quaid-i-Azam University Islamabad, Pakistan. For this purpose, before the experiment, healthy male rabbits (2500 ± 10 g) were kept in the primate facility with free access to food and water a day before the commencement of experiment. The rabbits were classified into two groups (n = 6). Group 1 was provided with CIP (25 mg) and Group 2 was given NH2-F127 SNEDDS (10 mL) through oral gavage needle. Afterward, the blood samples were extracted from marginal ear veins by sterile syringe in specified time intervals (0.5–24 h). Blood from each withdrawn sample was collected in 2 mL Eppendorf tubes containing anti-coagulant and plasma was extracted through centrifugation for 15 min at 4000 rpm, followed by the quantification of the drug (CIP) [27].

### 2.19. Statistical Analysis

T-test and ANOVA were conducted in statistical analysis for the assessment of the results and level of significance was less than 0.05.

## 3. Result and Discussion

The strategy behind the synthesis of novel NH2-F127 SNEDDS of ciprofloxacin is to enhance the advanced and highly specified targeted drug delivery against *Salmonella typhi*. The excessive amination of the pluronic can act as the carrier for targeting bacterial membrane by strongly attaching to it and disrupting its intracellular components, thus preventing multi bacterial drug resistance [28]. Therefore, highly precise and innovative selection of biocompatible polymer and *anti-salmonella* drug will help in circumventing oral, biological and cellular barriers against *S. typhi.*

### 3.1. Synthesis of Pluronic Amine Conjugate

**Figure 1:**
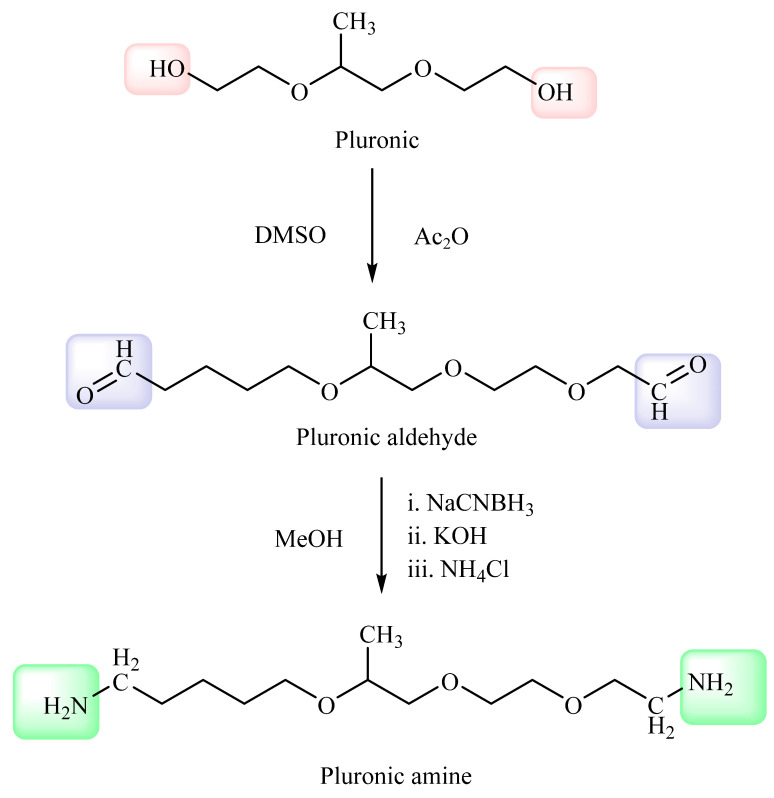
Conversion of pluronic into pluronic-amines.

Synthesis of pluronic-amines was carried out in two steps. Initially, the conversion of poloxamer into its aldehyde form confirmed by using Schiff reagent and then its attachment with amines, using reagents as shown in Figure 1. Moreover, the amounts of primary amine groups in the amidated pluronic were estimated by TNBS, respectively. According to the data, there were 454 ± 62 μmol primary amino groups per gram of polymer (mean ± SD; n = 3).

#### 3.1.1. Solubility Testing

**Figure 2:**
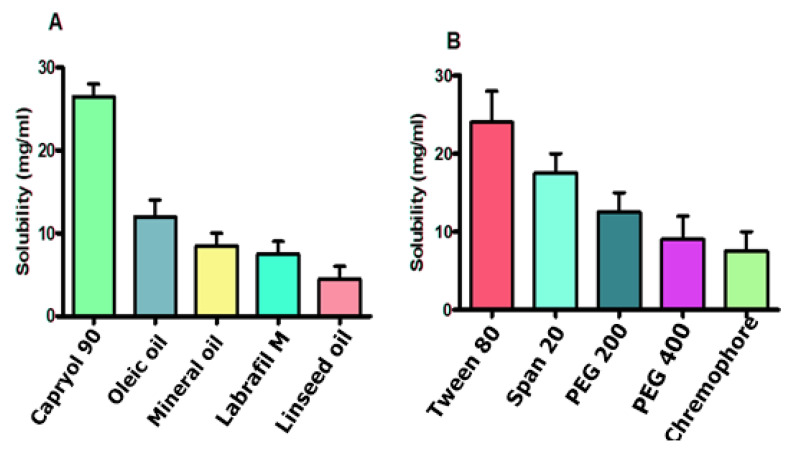
Solubility studies of drug in oils (**A**), surfactants, and co-surfactants (**B**).

Solubility testing of the CIP was conducted via preliminary screening of numerous excipients in terms of oils, surfactants, and co-surfactants. However, according to the results, it was determined that CIP SNEDDS based formulation requires Capryol 90, Tween 80, and Span 20 as suitable oil, surfactant and co-surfactant, respectively, as shown in Figure 2A,B. Moreover, from the literature and graphical representation, it is quite evident that CIP is also soluble in oleic and mineral oil, and Span 20 as surfactant and PEG 200 as co-surfactant.

#### 3.1.2. Pseudoternary Phase Diagram

**Figure 3:**
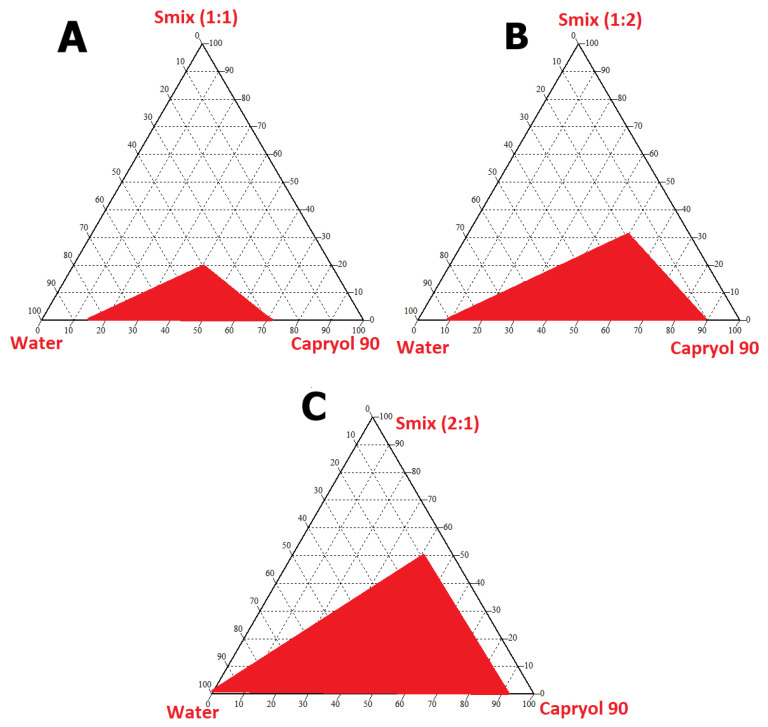
Ternary diagrams demonstrating red colored region indicating clear nanoemulsion region in various ratios (**A**–**C**).

Pseudoternary phase diagrams were constructed on the basis of selected suitable excipients (oil, surfactant, and co-surfactant) for the drug (CIP). However, for determining the nanoemulsion region, the specified ratio adjustment of surfactant/co-surfactant was quantified. The phase diagram constitutes oil, km/Smix ratio, and water in each corner with a cumulative 100%. In this study, the Km ratio of 1:1, 1:2, and 2:1 was selected. The red-colored region showed a nanoemulsion region and it was observed that it was maximum with an adjustable Smix ratio of 2:1 as shown in Figure 3. Ternary phase diagrams are indispensable in determining the best suitable surfactant as well as co-surfactant in formulating and optimizing SNEDDS.

#### 3.1.3. Formulation of Amidated Pluronic Loaded SNEDDS

The NH_2_-F_127_ loaded SNEDDS of CIP were formulated by adding adjustable quantities of the drug along with the polymer as identified by ternary diagrams into the blank SNEDDS formulation. NH_2_-F_127_ SNEDDS was tested via physicochemical characterization parameters and results showed thermodynamic stability, robustness to dilution, clarity for 48 h as well as the highest transmission as shown in Table 1. Moreover, the resulting optimized formulations were further tested for in vitro, ex vivo, and in vivo formulations.

#### 3.1.4. Size, SEM Analysis, Poly Dispersity Index, and Zeta Potential

**Figure 4:**
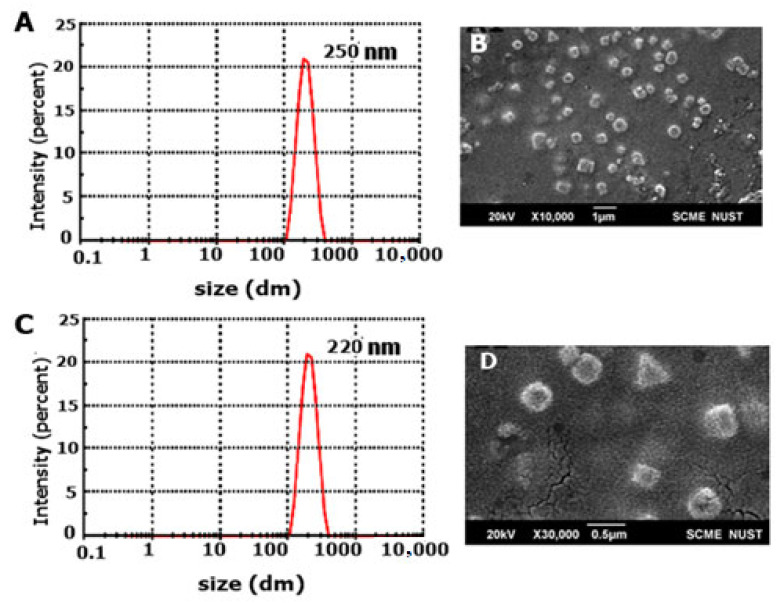
DLS graph of NH2-F127 SNEDDS formulation (**A**), Scanning electron micrograph of NH2-F127 SNEDDS formulation (**B**), DLS graph of F127 SNEDDS formulation (**C**), Scanning electron micrograph of F127 SNEDDS formulation (**D**).

The size of the NH2-F127 SNEDDS was found to be 250 nm with 0.3 PDI as confirmed by the dynamic light scattering (DLS) results and SEM results displaying uniformly dispersed particles as shown in Figure 4. Positive zeta potential owes to the inclusion of highly cationic NH2-F127. Moreover, the zeta sizing results of other SNEDSS formulations are shown in Table 2.

#### 3.1.5. Fourier Transformed Infrared Spectroscopy (FTIR) DSC

**Figure 5:**
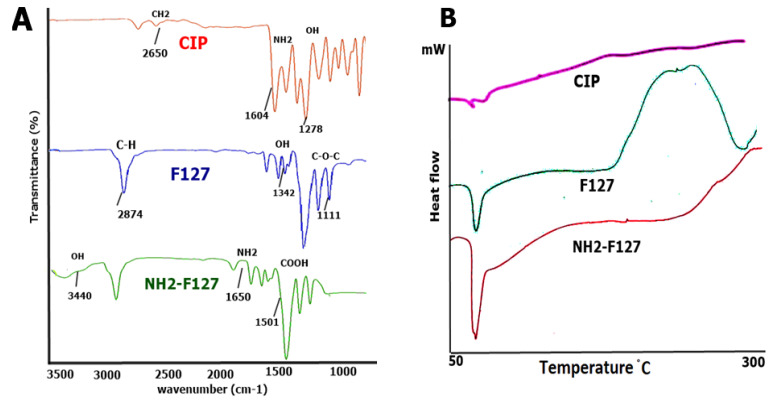
FTIR spectrum of CIP, F127, and NH_2_-F127 (**A**), DSC thermograms of CIP, F127, and NH_2_-F127 (**B**).

FTIR analysis was evaluated to determine the level of interaction between polymers and the drug. Results showed no interaction as shown in Figure 5A. FTIR spectrum of CIP showed stretching at 2650 cm^−1^ to confirm the presence of CH_2_ bonds. NH_2_ bonds were observed at a wavelength of 1604 cm^−1^. The characteristic OH group of CIP was visible at 1278 cm^−1^ bond stretching. In the case of pluronic, C-H stretching was observed at 2874 cm^−1^ along with OH stretching at 1342 cm^−1^. The characteristic peak of C-O-C was visible at 1111 cm^−1^ bond stretching. However, in the case of NH_2_-F127, OH and COOH peaks were observed at 3440 cm^−1^ and 1501 cm^−1^. The conjugation of the amino group to the pluronic was confirmed via a sharp peak of NH_2_ at 1650 cm^−1^ wavelength.

DSC of the CIP showed drying at 55 °C and degradation peak at 150 °C. Moreover, the DSC of F127 showed a distinctive drying polymer peak at 56 °C and a thermal degradation peak at 190 °C. However, the DSC of the NH2—F127 sample started denaturation at 55 °C, and degradation at 200 °C as shown in Figure 5B.

### 3.2. In Vitro Drug Release Study

**Figure 6:**
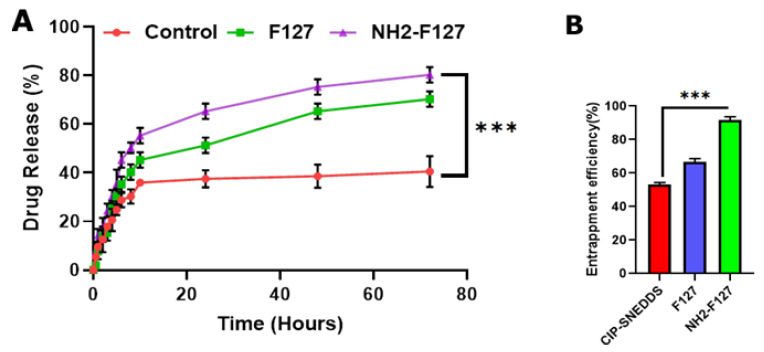
(**A**) In vitro drug release (**B**) drug entrapment of SNEDDS. The results are listed as mean ± S.D (n = 3). *** indicates *p* value ≤ 0.001.

*S. typhi* pathogen resides and proliferates in the phagosomes for systemic dissemination. Therefore, targeted delivery requires the sustained release of the drug in the phagosomes. For achieving targeted and sustained release, this study was carried on at pH 5.0 for mimicking the phagosomal pH for 72 h. The phagosomal drug release mechanism lies in the redox-triggered drug release. The drug release from the control group (CIP) initially showed a burst release of 30% within 24 h and maximum drug release was up to 40% in 72 h. F127 SNEDDS showed sustained drug release up to 60% in 72 h. As far as NH2-F127 SNEDDS were concerned, 10% drug was released in 10 h, 60% in 24 h, and up to 80% in 72 h with a substantial statistical difference (*p* ≤ 0.05) as shown in Figure 6A. The stable and sustained drug release was attributed to the protonation of the amino groups and steric stabilization of the polymers [29]. As far as kinetic modeling of the dissolution parameters is concerned, CIP showed first-order release showing an R^2^ value of 0.923. In the case of NH2-F127 SNEDDS, the Korsmeyer–Peppas drug model was followed with R^2^ values of 0.997 along with Fickian drug release with n ≤ 0.3 as shown in Table 3.

### 3.3. Entrapment Efficiency (%)

The entrapment efficiency of the drug in the SNEDDS was analyzed and the result confirmed the CIP maximum entrapment of up to 90% in terms of NH2-F127, while F127 and CIP-based SNEDDS showed entrapment up to 65% and 52% as shown in Figure 6B. Results concluded that maximum entrapment owed to the SNEDDS as a highly stabilized carrier system, and coating with NH2-F127 further increased its steric stabilization to encapsulate the drugs more effectively [29].

### 3.4. Mucopenetration Studies

**Figure 7:**
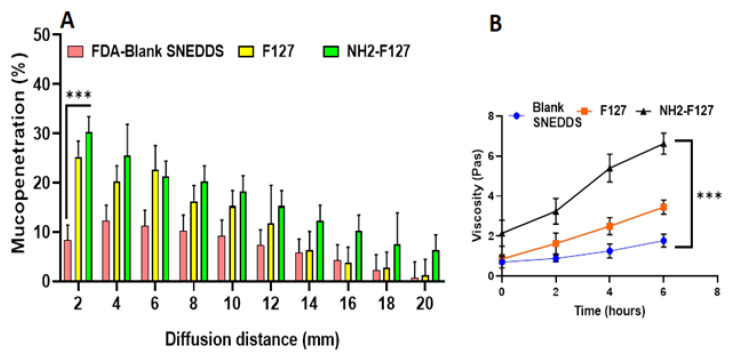
(**A**) Mucopenetration study of FDA labeled SNEDDS (**B**) mucoadhesive study of SNEDDS. The results are listed as mean ± S.D (n = 3). *** indicates *p* value ≤ 0.001.

Mucopenetration studies are basically mucus diffusion studies of the formulations. The study findings showed the maximum penetration of NH2-FI27 SNEDDS in the mucosal segments as compared to others, as shown in Figure 7A. The reason for increased mucosal diffusion is based on two important facts, i.e., the mucopenetration capability of amidated pluronic, aiding the formulation in reaching lower layers of the intestinal epithelium and overcoming anti-microbial poor penetration. Moreover, intestinal mucin bears a negative charge and hence attracts positively charged nanoparticles. The difference in the net charges between mucin and formulation is the leading source of mucopenetration. Moreover, from the literature review, it has been confirmed that amidated pluronic is rich in cationic ions, and these ions help in the improved cellular uptake and mucus diffusion [30].

### 3.5. Mucoadhesion via Rheological Synergism

A cone plate viscometer was used to determine the mucoadhesive nature of NH2-F127. Results concluded that NH2-F127 was two-fold more mucoadhesive as shown in Figure 7B as compared to the other formulations. NH2-F127 showed the strong binding of cationic rich polymeric nanoparticles with mucin, and ultimately strong binding with mucin resulted in increased retention and mucoadhesion [31]. Moreover, it is the matter of fact that mucosal retention capability of cationic NH2-F127 was extended as compared to that of negatively charged or amphoteric SNEDDS.

### 3.6. Hemocompatibility Assay

**Figure 8:**
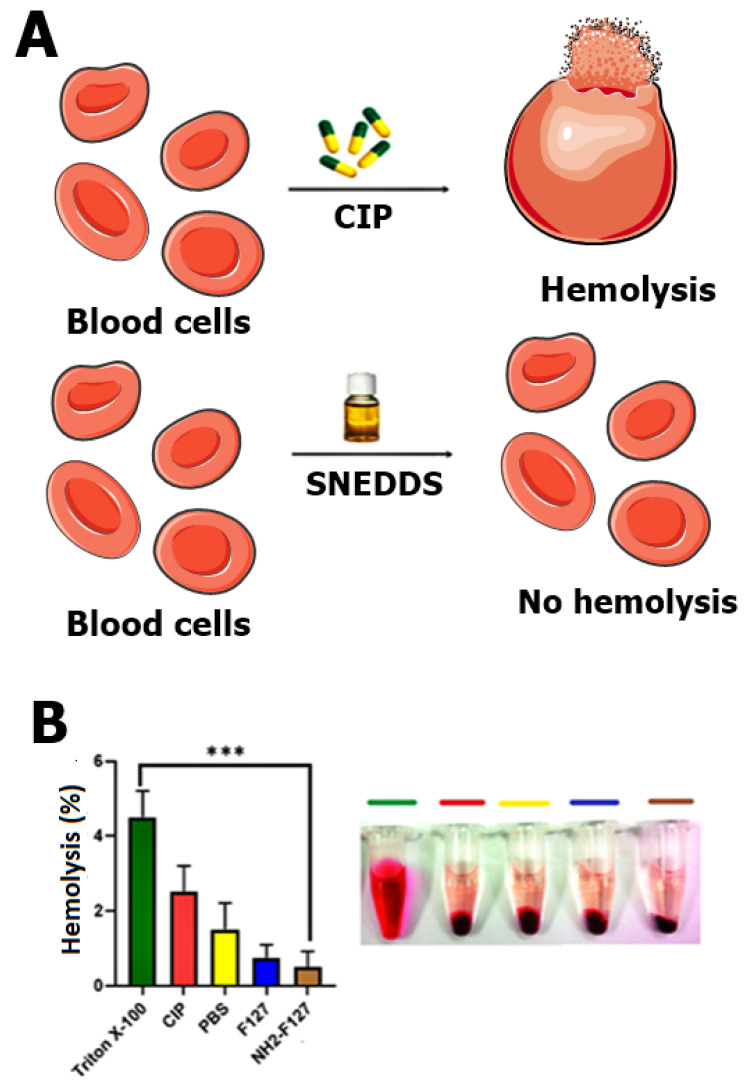
Schematic representation of hemolysis by treating with CIP and SNEDDS (**A**), evaluation of hemocompatibility (**B**). The results are listed as mean ± S.D (n = 3). *** indicates *p* value ≤ 0.001.

Polymeric NH_2_-F127 based SNEDDS were tested for determination of blood lysis to ascertain its hemocompatibility parameter. Positive control group to be determined was (Triton X-100 and blood) and negative control constitutes (PBS and blood). CIP SNEDDS CIP SNEDDS showed hemolysis, while NH2-F127 SNEDDS showed no visible signs of hemolysis (0.2%) as shown in Figure 8A. However, from results it can be interpreted that CIP SNEDDS showed 3% hemolysis, F127 SNEDDS showed 0.5% and NH_2_-F127 SNEDDS showed 0.2% hemolysis as shown in Figure 8B. The increased hemocompatibility of the synthesized SNEDDS was attributed to the inclusion of biocompatible and biodegradable polymer (NH_2_-F127).

### 3.7. In Vitro Lipolysis Studies

**Figure 9:**
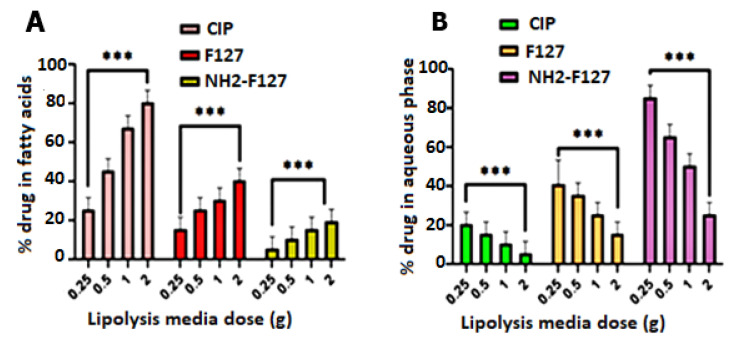
Lipolysis studies for evaluating drug % age in the undigested fatty acids (**A**) and aqueous phase (**B**). *** indicates *p* value ≤ 0.001.

In vitro lipolysis studies showed the SNEDDS potential in penetrating intestinal mucus, followed by absorption of the drug (CIP) in various layers of lipolysis media. It is obvious from the results that NH2-F127 SNEDDS showed maximum solubilization in the aqueous phase as compared to the undigested fatty acids layer of the lipolysis medium. Lipolysis has an inverse relation to lipid load as shown in Figure 9. Moreover, CIP maximum solubilization and recovery in the aqueous phase is due to the presence of NH2-F127 SNEDDS in the aqueous layer.

### 3.8. In Vitro Cell Cytotoxicity of S. typhi in Macrophage RAW 264.7 Cells

**Figure 10:**
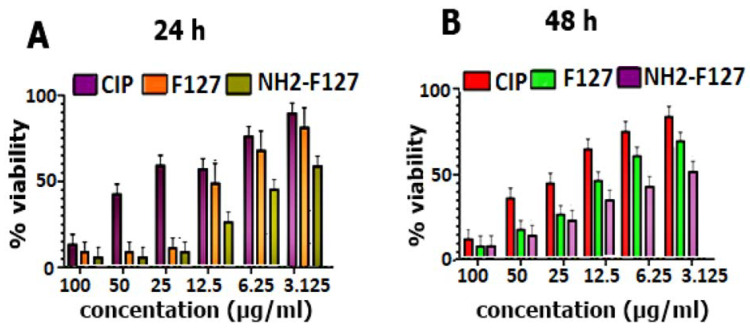
In vitro cytotoxicity evaluation of pure CIP and SNEDDS formulations on *S. typhi* infected macrophages (**A**) for 24 h (**B**) for 48 h. The results are represented as mean ± S.D (n = 3).

In vitro cell cytotoxicity of Salmonella typhi in macrophage RAW 264.7 cells was evaluated to determine and compare the anti-salmonella typhi potential of advanced SNEDDS formulation via time and concentration-dependent inhibitory effect (IC50 value). There was a significant difference (*p* < 0.01) between the cytotoxic potential of CIP suspension and CIP-SNEDDS as shown in Figure 10. The IC50 value was lowest for the NH2-F127 and F127 as compared to CIP-SEDDS and blank SEDDS as shown in Table 4. NH2-F127 showed a minimum IC50 value owes toward the synergistic antibacterial effect, steric stabilization, amination, non-toxic nature, and increased uptake features of for intracellular drug delivery [32].

### 3.9. Anti-Bacterial Activity

**Figure 11:**
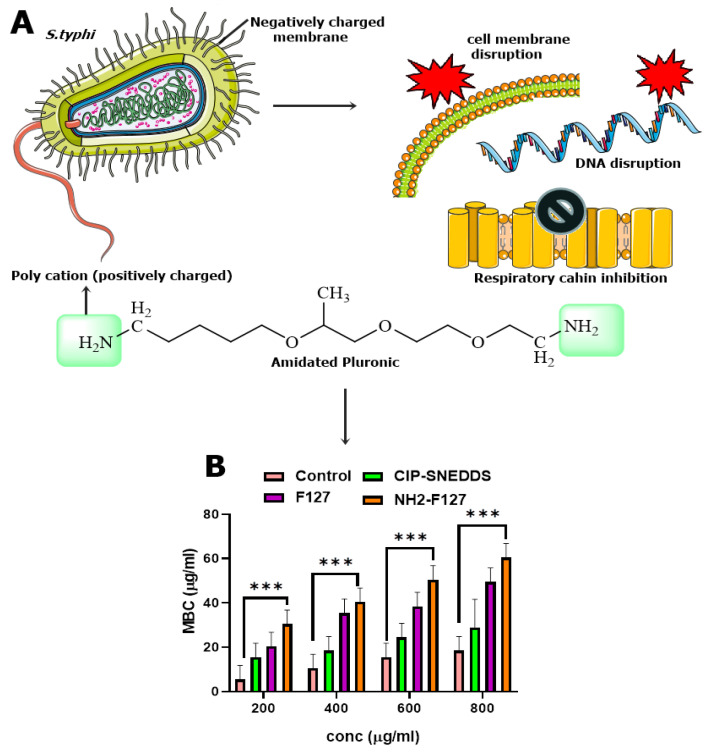
Mechanism of interaction of positively charged polymer with negatively charged *S. typhi* membrane, leading to growth inhibition and disruption of *S. typhi* (**A**), MBC of SNEDDS formulation (**B**). *** indicates *p* value ≤ 0.001.

Antibacterial activities of the formulations were determined and results showed that NH2-F127 SNEDDS have great anti-microbial potential as depicted in minimum bactericidal concentration (MBC) as shown in Figure 11B. The antibacterial mechanism by pluronic-amines is an interesting enigma as conjugation of the –NH- group leads to the intracellular penetration and burst of cationic ions release in the bacterial cells as well as infected macrophages to diminish the bacterium as shown in Figure 11A. It can also be explained as the amino groups’ protonation leading to strong electrostatic interactions and increased contact time with negatively charged bacterial membranes. Prolonged contact time between nanoformulations and the bacterial membrane is the leading source of bacterial destruction, in terms of promoting an antibacterial action [33].

### 3.10. SNEDDS Uptake Studies

**Figure 12:**
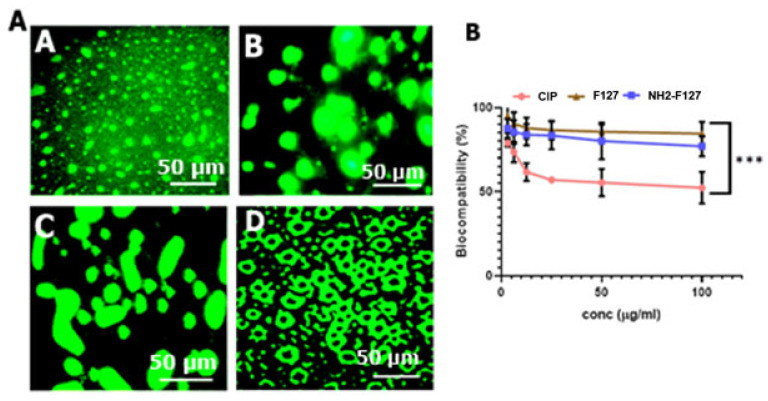
(**A**). FITC labeled blank (A), CIP SNEDDS (B), F127-SNEDDS (C), and NH2-F127 SNEDDS (D) showing fluorescence. Scale bars (50 µm) (**B**) biocompatibility of the SNEDDS. Results are listed as mean ± S.D (n = 3). *** indicates *p* value ≤ 0.001.

Macrophage uptake studies confirmed the highest fluorescence of NH2-F127 SNEDDS treated macrophages as compared to that of blank SNEDDS as shown in Figure 12A. High fluorescence imaging indicated the amine group penetrating capability and maximum accumulation as well as electrostatic interaction with macrophage receptors.

### 3.11. Biocompatibility Studies

Biocompatibility studies of the SNEDDS formulations with varying concentrations (low to high levels) showed 90% viability of the cells when treated with NH_2_-F127, even at maximum drug proportion as shown in Figure 12B. This increased cell viability owes to the biocompatible nature of the polymers [11].

### 3.12. Biofilm Elimination Assay

**Figure 13:**
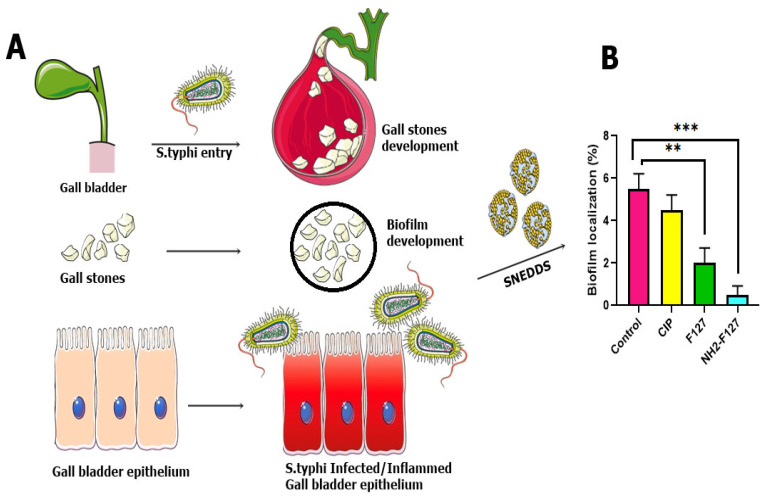
Schematic representation of biofilm development (**A**), biofilm localization assay (**B**). Results are listed as mean ± S.D (n = 3). *** indicates *p* value ≤ 0.001, ** indicates *p* value ≤ 0.01.

**Figure 14:**
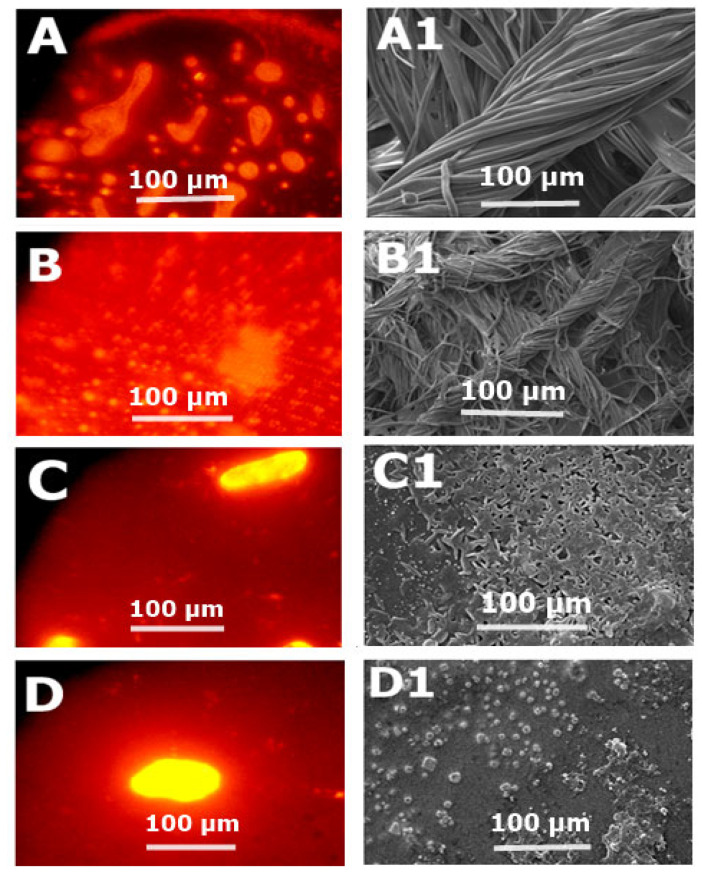
Fluorescence imaging of rhodamine-labeled *S. typhi* biofilms indicating the control (**A**), CIP (**B**), F127 (**C**), and NH2-F127 (**D**). SEM analysis of the biofilm’s reduction in step-wise process indicating the control (**A1**), CIP (**B1**), F127 (**C1**), and NH2-F127 (**D1**).

Antimicrobial drug resistance has facilitated the pathogens such as *S. typhi* to develop biofilms. Biofilms optical density values concluded that NH_2_-F127 SNEDDS were efficient in the dispersion/elimination of the co-localized biofilms. NH_2_-F127 SNEDDS resulted in a marked reduction in biofilm localization, i.e., 0.04% as compared to the 1.7% of the control group, respectively, as shown in Figure 13B. Fluorescence imaging of the biofilm reduction is shown in Figure 14A. The proficient biofilm dispersion is owing to the strong antibacterial action and disruption of the *S. typhi* bacterium by the accumulation of cationic ions [34].

### 3.13. Biofilms Dispersal Assay in Gall Bladder

*S. typhi* infection proliferates and disseminates from the intestines toward the gall bladder and systemic circulation. Infection in the gall bladder leads to the impregnation of biofilms in the form of gall stones. Gall stone biofilms SEM analysis showed a marked reduction in the aggregation as compared to the CIP SNEDDS and control with statistical significance (*p* ≤ 0.05) as shown in Figure 14.

### 3.14. In Vitro Survival Assay

**Figure 15:**
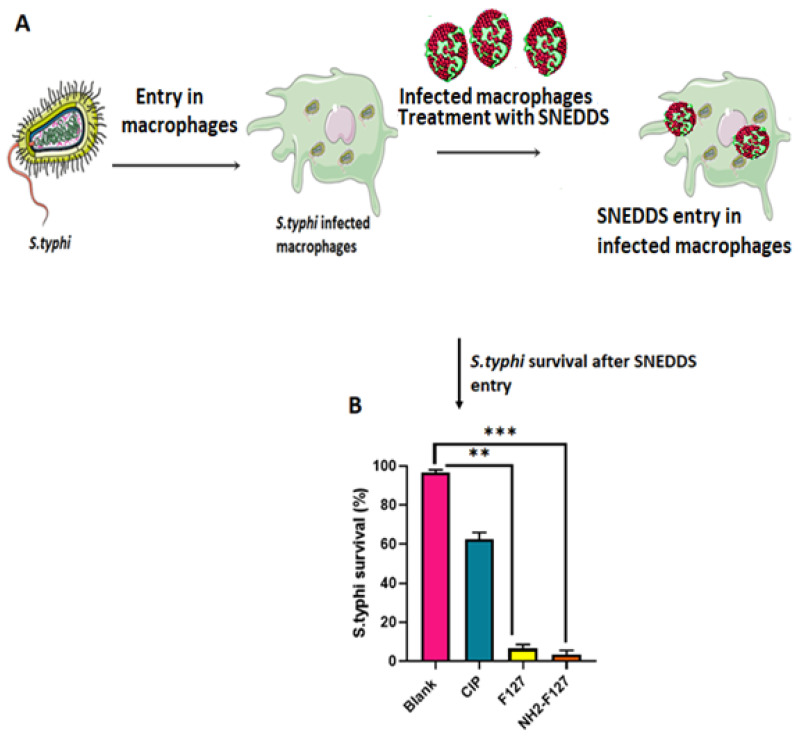
Schematic representation of the *S. typhi* infection in macrophages (**A**) in vitro survival of NH2-F127 treated *S. typhi* in RAW 264.7 macrophages (**B**) *** indicates *p* value ≤ 0.001, ** indicates *p* value ≤ 0.01.

The *S. typhi* survival in the infected macrophages was markedly reduced by treatment with NH_2_-F127 as compared to a simple drug (CIP). *S. typhi* viability in the macrophages treated with NH2-F127 after 24% was only 2% as compared to 95% viability in CIP as shown in Figure 15 The proficient eradication and targeted killing of *S. typhi* is associated with enhanced phagosomal uptake by scavenger cells and targeted strong anti-bacterial action [35].

### 3.15. In Vivo Pharmacokinetics

**Figure 16:**
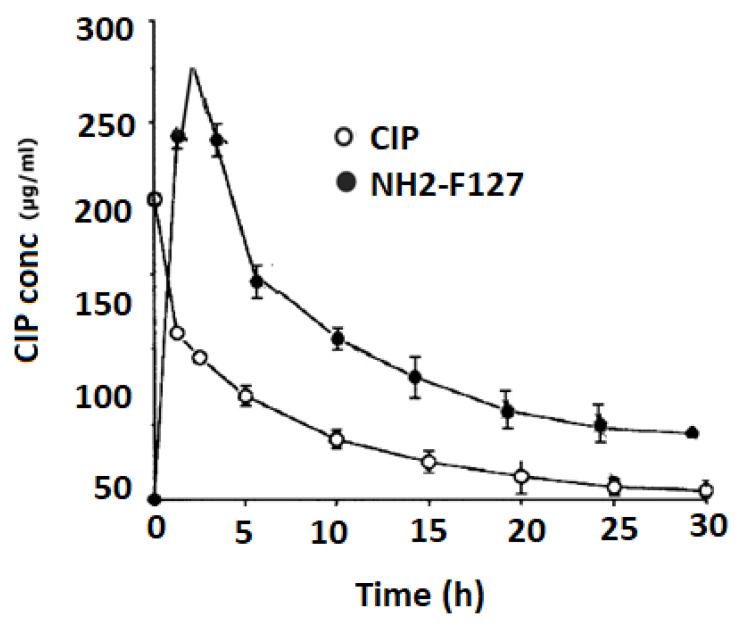
In vivo pharmacokinetics of CIP and NH2-F127.

In vivo pharmacokinetic distribution of CIP and NH2-F_127_ SNEDDS are shown in Figure 16. Results showed that NH2-F127 SNEDDS showed improved half-life, T max, Cmax, and AUC in terms of CIP quantification as compared to the CIP suspension. The results of the following parameters are mentioned in Table 5.

The highly improved under curve (AUC) value showed the accomplishment of SNEDDS in transportation of hydrophobic drugs into the systemic circulation

## 4. Conclusions

NH2-F127 SNEDDS were examined as prospective transporters for CIP, which possesses mucosal barriers in reaching intestinal epithelial layers. The NH2-F127 SNEDDS resulted in privileged internalization by *S. typhi*-infected macrophages, followed by targeted killing and eradication. The sustained drug release from NH2-F127 SNEDDS showed efficient anti-salmonella treatment with minimal antibacterial drug (CIP) doses. Additionally, NH2-F127 SNEDDS showed biocompatibility, hemocompatibility, and biofilm reduction. SEM illustrations of the *S. typhi*-impregnated gall stones showed marked reduction and dispersal of biofilms as compared to non-other SNEDDS. Collectively, the present results designate that the inventive NH2-F127 SNEDDS seems to be an intriguing carrier system for better therapeutic outcomes against *S. typhi*.

## Figures and Tables

**Table 1:** Saturation solubility, clarity, cloud point, transmittance (%), and emulsification time of SNEDDS formulation.

SNEDDS Formulation	Saturation Solubility (mg/mL)	Clarity	Cloud Point (°C)	Transmittance (%)	Emulsification Time (s)
CIP	60.5	Turbid	60	80.4	50
F127	75.4	Clear	65	89.7	20
NH_2_-F127	95.3	Clear	84	94.6	10

**Table 2:** Results of size and zeta-potential measurements. The results are listed as mean ± S.D (n = 3).

SNEDDS Formulation	Size (nm)	Zeta Potential (mV)	PDI
CIP-SNEDDS	180 ± 4.1	+21.4 ± 4.6	0.5 ± 1.4
F127	220 ± 2.5	+18.0 ± 2.5	0.3 ± 2.6
NH_2_-F127	250 ± 1.7	+39.0 ± 3.2	0.2 ± 3.1

**Table 3:** Kinetic models to evaluate the drug release mechanism from SNEDDS.

Kinetic Models	Zero Order Ct = C0 + k0t		First Order logQ0 + Kit/2.3		Korsmeyer–Peppas F = (M t M) = K m · t n		Higuchi f1 = Q = KH√t	
Formulations	R^2^	K^0^	R^2^	K_1_	R_2_	n	R_2_	KH
CIP	0.550	22.90	0.923	0.677	0.5370	0.502	0.7310	43.70
F_127_	0.5227	3.77	0.9358	0.237	0.9785	0.345	0.8678	16.310
NH2-F127	0.5231	4.29	0.9465	0.160	0.997	0.235	0.8920	15.45

**Table 4:** IC_50_ values of pure CIP, non-polymeric and polymeric SNEDDS from cell cytotoxicity data. The values are listed as mean ± S.D (n = 3).

Formulations	IC_50_ Value (µg/mL)	
	After 24 h (µg/mL)	After 48 h (µg/mL)
**CIP**	20 ± 4.9	12.12 ± 2.2
**F_127_**	6.51 ± 1.4	5.62 ± 1.6
**NH_2_-F_127_**	3.58 ± 0.9	2.38 ± 1.4

**Table 5:** Evaluation of the in vivo pharmacokinetic parameters.

Parameter	CIP	NH2-F_127_
t_1/2_ (h)	4 ± 0.9	18 ± 1.9
T max (h)	1 ± 1.9	4 ± 0.8
C max (µg/mL)	50.50 ± 0.5	220.15 ± 0.4
AUC 0-t (μg × h/mL)	200.75 ± 0.6	1662.83 ± 0.3
AUC 0-inf (μg × h/mL)	600.84 ± 0.1	3124.50 ± 0.1
AUMC 0-inf (μg × h/mL)	900.65 ± 0.7	4001.5 ± 0.7

## Data Availability

Data will be provided on demand.
